# Partnerships and Collaborations: The Right Alliances for Clinical Trials in Africa

**DOI:** 10.1200/JGO.19.00194

**Published:** 2020-07-02

**Authors:** Olusola Solarin, Sulma I. Mohammed, Ntokozo Ndlovu, Verna Vanderpuye, Victoria Olaiya

**Affiliations:** ^1^ Savante Consulting, Lagos, Nigeria; ^2^ Department of Comparative Pathobiology, Purdue University Center for Cancer Research, West Lafayette, IN; ^3^ Department of Radiology, College of Health Sciences, University of Zimbabwe, Harare, Zimbabwe; ^4^ Center for Radiotherapy Oncology and Nuclear Medicine, Korle-Bu Teaching Hospital, Accra, Ghana; ^5^ Synteract, Ely, Cambridgeshire, United Kingdom

## Abstract

Africa attracts < 1% of all trials conducted around the world. The implication is that proof of safety and efficacy in Africans is lacking for a lot of new therapies. The sizeable proportion of approximately 20% of the global population that Africa represents largely does not have empiric data to support use of new therapies in a population with a distinct genetic and racial profile. Beyond the imperative of evidence-based interventions, Africans carry a disproportionately heavy burden of certain diseases, including prostate cancer, sickle cell anemia, and malaria. It therefore provides opportunity for efficient recruitment of participants for trials for such diseases. However, this advantage has not convinced sponsors to carry out clinical trials in Africa. India and China each have roughly the same population size as Africa, but each presents just one regulatory jurisdiction for clinical trials. Africa has 54 countries, and a sponsor would theoretically need to file 54 different applications to cover the entire continent. Collaboration and partnership among all stakeholders in the clinical trial ecosystem will reduce the burden on sponsors and make Africa competitive as a destination for clinical trials. Collaboration among national regulatory agencies will enable Africa to be treated as one regulatory jurisdiction and reduce administrative burden. Sites and researchers can partner to improve quality, attain necessary certifications, and increase overall efficiency. Central to all of these are clinical research organizations that can coordinate and work across borders to make clinical trial projects seamless. Ultimately, patients will benefit as quality of clinical practice improves and access to new therapies is enhanced.

## INTRODUCTION

Africa is host to > 18% of the world’s population and 3% of the global pharmaceutical market, but it attracts < 1% of registered clinical trials.^1^ Despite the advantage Africa offers with its unique and diverse genetic pool and ease of recruiting trial participants in pathologies like sickle cell anemia, prostate cancer, and some infectious diseases, Africa is still disproportionately poorly represented in clinical research. In this article, we will examine reasons for this. We argue that collaboration among stakeholders in the clinical trial ecosystem can improve the attractiveness of Africa as a clinical trial destination for sponsors. Regulatory agencies, researchers/clinicians, research sites, and sponsors can play key roles in improving the ease of conducting trials in Africa.

**CONTEXT**

**Key Objective**
This article highlights how partnerships and collaborations among stakeholders can help attract clinical trials to Africa based on local experience and literature review.
**Knowledge Generated**
These partnerships must involve regulatory agencies, clinicians, researchers, sites, patients, and sponsors as identified stakeholders in the clinical trial ecosystem. Development of certification and continuous training for players involved is essential to support conduct of more clinical trials in Africa.
**Relevance**
One solution to enable the standardization and uniformity necessary for the conduct of clinical trials in Africa may be a single regulatory jurisdiction for the continent, a framework for the achievement of which is proposed in this article.

## 54 COUNTRIES, 54 MARKETS, 54 REGULATORY JURISDICTIONS

Each of Africa’s 54 countries represents a regulatory jurisdiction for clinical trials and market for health care products. Each of these countries competes for clinical trials with India and China, each of which has roughly the population as Africa but offers sponsors one regulatory jurisdiction and, in the case of China, a much bigger market. Sponsors are likely to find it easier to deal with a single regulatory agency, as in each of these two countries, than with the 54 that Africa presents.

Also complicating the bureaucratic challenge of conducting trials in Africa is the fact that national regulatory agencies are at different maturity levels.^2^ Therefore, the case for partnership and collaboration to support clinical trials in Africa is a compelling one. Collaboration among Africa’s national regulatory agencies (NRAs) for medicines could improve competitiveness for clinical trials, although planning and implementation of this remain challenging.

## COLLABORATION AMONG RESEARCHERS IN AFRICA

Aided by advances in information-sharing technologies and social media platforms, such as ResearchGate,^3^ collaborative research is already in practice and is evidenced by the rapid growth in the number of authors in a publication. Health research assumed collectively by more than one researcher/clinician within the same department, or across academic departments in an institution or in different academic institutions or industries, has also become more common across countries. It provides opportunity for collaborators to share expertise and resources and expand their skills and worldviews on health issues. It may yield improved and unique data that can be used to design specific drugs and targeted medical interventions.

Successful collaboration is built on trust, understanding, and selflessness. It entails putting the interests of the team and participants first. It provides an opportunity for putting expertise and resources together to advance medical knowledge for appropriate intervention.

Global collaborative health research is essential to addressing complex diseases, such as cancer, a growing problem in Africa. It requires multidisciplinary expertise. Collaboration among social workers, physicians, nurses, scientists, and industries has proven to be essential in conducting successful clinical research. This collaboration benefits not only the patients and the science behind treatment, but also individual researchers mentoring younger ones to develop scholarly attributes. Cross-cultural collaboration promotes healthy interaction among scientists from different countries, ensuring scientists and oncologists gain a broader worldview of the burden of cancer, social interplay, and management. It goes without saying that ethically based collaboration can yield better scientific data, increase the capacity for research, improve funding, empower partners, expose collaborators from different countries to different cancer biologies, and contribute to the personal and professional development of all involved.

Despite its many benefits, collaborative research may have setbacks. The heterogeneity of the sociocultural backgrounds of scientists involved, resources (time and money) contributed, professional reputations of researchers, and attributions of credit (authorship) may be sources of tension.^4^ A culturally unfit and ill-conceived collaboration can destroy trust and breed hostility toward future collaborations.

## COLLABORATION AMONG SITES TO HARMONIZE TREATMENT GUIDELINES AND BOOST EFFICIENCY

Various models of collaborative partnerships between sites within a country or region or between regions have their own merits and pitfalls. Advantages include addressing of common areas of interest, transfer of knowledge and skills, local mentorship of young investigators, ease of recruitment of trial participants, provision of a wider range of clinical judgments, and provision of local evidence for the subsequent generalization of trial findings.

For many African clinical trials, the scenario is usually that of a single or multiple sites collaborating with an international site. Collaboration among African sites seems to be rare. Many reasons have been postulated for this, including lack of funding and limited research capacity. High-income countries fill the gap by conceptualizing, coordinating, and funding African studies, and the resultant effect at times is the promotion of trials that are not of critical importance to Africa’s health care problems.^5-7^

Most African countries have similar challenges in cancer management, and there is a dearth of local evidence-based solutions and approaches. More collaborations among African sites should be encouraged to facilitate the development of local treatment guidelines and comprehensive national cancer control programs. Collaboration among sites provides a platform for plugging existing gaps in an efficient and timesaving manner and expediting the generation of extensive data. This was successfully demonstrated in a study performed in one African country among 12 sites in one region. The collected data included trends in HIV prevalence, HIV incidence, HIV-associated and all-cause mortality, sexual risk behaviors, and health-seeking behaviors. The investigators were able to measure the effects of prevention and treatment programs on the HIV epidemic and generalize them to the whole country.^8^ In another publication, a review of collaboration between HIV care centers and cancer centers was carried out. The findings demonstrated the merits of integrating cancer research with established HIV programs to obtain timely data about the incidence and burden of cancer in HIV-infected persons in Africa.^9^

Collaboration among sites also generates problem-solving solutions through standardization and adherence to case definitions, clinical procedures, laboratory methods, and specimen collection techniques.^10^ Broader collaboration corrects for the differences in practice norms that are inevitable across countries and continents. Addressing and defining the standard of care at various trial sites are important. Study data are credible only if the quality of collection and processing of data are standardized across sites, especially in resource-constrained situations. Challenges such as the lack of qualified personnel and experience in designing software tools and complex databases are common in Africa. These challenges can be effectively resolved if identified earlier on in the design of studies.^11,12^ The recognition of potential harm in sharing data among sites has been explored. One study explored the possibility of the use of genomic data in ways that could cause perceived harm to ethnic groups involved in studies. The potential for abuse may arise where there is sharing of genomic data outside the original group collaborating. Trust is important for such collaborations to thrive, and safeguarding against such misuse is important.^13^

Recognition and integration of work culture are important for successful collaboration across sites, especially in Africa, where diverse ethnicities make regional as well as international collaborations intriguing.^14^ To this effect, international clinical electives have been shown to be effective in preparing young potential investigators to receive cross-cultural educational and gain research skills to equip them for future collaborative work.^15^

## COLLABORATING TO ASSURE QUALITY OF OUTCOMES ACROSS BORDERS

The paucity of clinical trials in Africa compared with other low- and middle-income countries is also blamed on the lack of stringent quality assurance measures and training to execute the processes necessary to maintain quality of outcomes. Compared with high-income countries, many institutions in Africa do not have training modules in clinical trials for frontline health practitioners, resulting in the major activities of the required processes being outsourced to international partners.^16^ Many trials involve several interlacing disciplines and therefore require that each aspect has the ability to contribute efficiently and appropriately. Best practices to improve clinical trials in Africa involve ensuring standardization of processes, starting from capacity building and continuing through liaison with partners conducting high-quality clinical trials and thereby improving quality. The best way to ensure standardization and uniformity across the continent is to have processes verified by universally acclaimed certification. This may involve several exchange visits, human resource training, and equipment upgrades. Principal investigators or key players must be encouraged to obtain clinical research associate certification, which will include a course on good clinical practice offered by an accredited institution, usually involving an examination. Institutions must be certified to ensure provision and maintenance of required infrastructure, and this should be an initial focus of engagement with international collaboration to develop capacity. Formation of regional consortiums must be encouraged to improve quality, scale cultural barriers, and identify regional certification opportunities.

Currently, mandatory courses for certification are fundamentals of clinical trials, human participant safety, and regulatory requirements for medical devices and pharmaceutical products. However, bioethics, health law, and data management are parts of good clinical practice and should also be mandatory, because they are critical to initiation, enrollment, reporting, and outcomes.^17,18^ A mandatory requirement to register clinical trials in international, national, or regional platforms will also increase proficiency and transparency and serves as a form of certification to improve standards of conducting clinical trials. Indeed, many will strive for listing in regional or international registries with international accreditation. This module was instituted in Italy for phase I trials and resulted in increased standards and increased requests to obtain accreditation to conduct clinical trials in this category.^19^ WHO has developed guidelines for clinical trials registries, which could be embraced as a template by sub-Saharan African institutions.^20,21^ A retrospective study from the United States demonstrated improved standards after accredited good practice certification in four randomized trials.^22^

## PAYERS, CONTRACT RESEARCH ORGANIZATIONS, AND PHARMACEUTICAL COMPANIES

Unlike many high-income countries, private insurance does not play a dominant role in health care in Africa. Except for South Africa, the main payer is the government. It is prudent to recognize the emerging role of payers in the trajectory of the clinical trials landscape, because they, as with other stakeholders, will enable end-user access to these new interventions. Early collaboration will ensure minimal waste of resources, prioritize needs, formulate research questions, and enhance cost effectiveness of new innovations. This will help reduce financial losses and health care burden as a result of adverse effects of new interventions.

In recent years, there has been an increase in the number of biopharmaceutical companies using the services of contract research organizations (CROs) and other clinical trial service providers for the delivery of their research and development operations. In a research and market report published in June 2015, it was mentioned that by 2020, an estimated 72% of all clinical trials will be outsourced to CROs^23^; this is approximately three fourths of clinical trials.

A CRO provides research and clinical trial support services to the pharmaceutical, biotechnology, and medical device industries as well as to academic institutions, foundations, government organizations, and hospitals on a contract basis. An individual, company, institution, business, organization, or group of organizations that contracts with a CRO is called a sponsor.

Major pharmaceutical companies have a presence in Africa and are in partnerships with local businesses for support in navigating the diverse and heterogeneous market of > 50 countries. The pharmaceutical industry is increasingly looking toward emerging markets, including Africa, for drug development and basic research. It is estimated that the pharmaceutical industry is expected to invest $45 billion on the continent by 2020. Countries like Egypt and South Africa are home to 75% of the 8,897 clinical trials in Africa as a result of their comparative economic strength, followed by Tunisia, Kenya, Uganda, Tanzania, Zambia, and Burkina Faso, documenting at least 200 trials in the year 2019.^24^ These countries are striving for health improvements through strategic investments and promotion and facilitation of clinical research.

Pharmaceutical companies will often partner with technology organizations and CROs to set up and run their clinical trials. The CROs have access to local knowledge; they work with investigators and local clinical research teams in the identification and recruitment of patients, collection and analysis of patient data, and assessment of patient safety as regards adverse effects.^25^ There are global CROs with offices in Africa, and there are also locally established CROs. These organizations often work together in the delivery of clinical trial services.

CROs fall into different types. Preclinical CROs, which perform testing of drugs or medical devices before the compounds or devices go on to the human or clinical phase to ensure safety and effectiveness. Clinical CROs specialize in patient recruitment, site selection, monitoring, medical writing, regulatory preparation of applications to ethics committees and regulatory authorities, data management, and other clinical trial services.

CROs understand the local climate best suited for business with related economic outcomes for their investors, owners, and shareholders. For a successful CRO-sponsor collaboration, it is necessary for the sponsor to choose a CRO that will be an effective partner. This collaboration can be both rewarding and rich if it is well established. For the sponsor, outsourcing clinical trials via CROs has the potential to reduce costs and provide access to local knowledge and local expertize (country experts) in various geographic markets, thereby giving sponsor a global reach.

The following are keys to a successful CRO-sponsor collaboration: defining common goals up front, engaging in shared decision making, and establishing clear communication and practical project management plans as well as secure cloud platforms to be used for data sharing by the CRO and sponsor; defining what information is to be communicated during the study, the frequency of communications, and which issues require escalation^26^; and establishing the key performance indicators and how performance will be measured against set goals. In addition, an effective two-way communication process should be in place. These practices will create an atmosphere for positive collaboration that is beneficial to the partners and will help to strengthen the CRO-sponsor partnership.

The African Union is supporting the creation of an African medicines agency to coordinate the regulation of medicines, including clinical trials all over Africa. In 2006, the African Vaccine Regulatory Forum (AVAREF), a network of NRAs and ethics committees, was established by WHO to strengthen regulation, initially of vaccines, but lately, the platform has been extended to other medicines, and a joint review platform has been developed that sponsors can leverage to have their applications reviewed simultaneously by AVAREF member countries. It is a platform that has been used to review some clinical trial applications, especially during emergencies, and working documents and guidelines have been developed for harmonization within the continent to enable sponsors to develop a single application package (in case of multinational clinical trials) for review.

In conclusion, we believe that collaboration among clinicians, researchers, sites, and regulatory agencies is essential to making Africa an attractive destination for clinical trials. This, alongside quality certification of personnel, sites, and process, will win the trust of sponsors and CROs. All these moving parts must run on a framework that will make research seamless and outcomes trusted.

The first recommendation is for Africa to present just one regulatory jurisdiction to sponsors. The feasibility and applicability of this must be assessed with the diversity of practice within the continent of Africa in mind. Good clinical practice guidelines, training modules in clinical trials, and site checklists must be coordinated, with mutual recognition of inspections by different NRAs to reduce costs and bureaucratic burdens. To ensure standardization and uniformity across the continent, processes must be verified by universally acclaimed certification.

## References

[1] Novotny M: As clinical trials go international, operations go digital. http://www.appliedclinicaltrialsonline.com/clinical-trials-go-international-operations-go-digital

[2] Branigan D: WHO benchmarking tool made “more transparent” in evaluating regulatory authorities. https://www.healthpolicy-watch.org/who-benchmarking-tool-made-more-transparent-in-evaluating-regulatory-authorities/

[3] Wray BK The epistemic significance of collaborative research Philos Sci 69 150 168 2002

[4] Chetwood JD Ladep NG Taylor-Robinson SD Research partnerships between high and low-income countries: Are international partnerships always a good thing? BMC Med Ethics 16 36 2015 26017015 10.1186/s12910-015-0030-z PMC4447022

[5] Swingler GH Pillay V Pienaar ED et al International collaboration, funding and association with burden of disease in randomized controlled trials in Africa Bull World Health Organ 83 511 517 2005 16175825 PMC2626288

[6] Reddy P Desai R Sifunda S et al “You travel faster alone, but further together”: Learning from a cross country research collaboration from a British Council Newton Fund grant Int J Health Policy Manag 7 977 981 2018 30624871 10.15171/ijhpm.2018.73 PMC6326641

[7] Morgan JP Isyagi M Ntaganira J et al Building oral health research infrastructure: The first national oral health survey of Rwanda Glob Health Action 11 1477249 2018 29860930 10.1080/16549716.2018.1477249 PMC5990941

[8] Gregson S, Mugurungi O, Eaton J, et al: Documenting and explaining the HIV decline in east Zimbabwe: The Manicaland general population cohort. BMJ Open 7:e015898, 2017 10.1136/bmjopen-2017-015898 PMC5639985 28988165

[9] Mbulaiteye SM Bhatia K Adebamowo C et al HIV and cancer in Africa: Mutual collaboration between HIV and cancer programs may provide timely research and public health data Infect Agent Cancer 6 16 2011 22004990 10.1186/1750-9378-6-16 PMC3223125

[10] Crawley J, Prosperi C, Baggett HC, et al: Standardization of clinical assessment and sample collection across all PERCH study sites. Clin Infect Dis 64:S228-S237, 2017 (suppl 3) 10.1093/cid/cix077 PMC5447838 28575355

[11] Van den Broeck J Mackay M Mpontshane N et al Maintaining data integrity in a rural clinical trial Clin Trials 4 572 582 2007 17942472 10.1177/1740774507084106

[12] Forster M Bailey C Brinkhof MWG et al Electronic medical record systems, data quality and loss to follow-up: Survey of antiretroviral therapy programmes in resource-limited settings Bull World Health Organ 86 939 947 2008 19142294 10.2471/BLT.07.049908 PMC2649575

[13] de Vries J Williams TN Bojang K et al Knowing who to trust: Exploring the role of ‘ethical metadata’ in mediating risk of harm in collaborative genomics research in Africa BMC Med Ethics 15 62 2014 25124199 10.1186/1472-6939-15-62 PMC4146449

[14] van der Wiel R Challenging perceptions of disciplinary divide: An ethnographer’s experience of collegiality, collaboration and crisis Med Humanit 44 e2 2018 30482823 10.1136/medhum-2018-011476

[15] Monroe-Wise A Kibore M Kiarie J et al The Clinical Education Partnership Initiative: An innovative approach to global health education BMC Med Educ 14 1043 2014 25547408 10.1186/s12909-014-0246-5 PMC4335420

[16] Bhatt A Quality of clinical trials: A moving target Perspect Clin Res 2 124 128 2011 22145122 10.4103/2229-3485.86880 PMC3227329

[17] Alemayehu C, Mitchell G, Nikles J: Barriers for conducting clinical trials in developing countries: A systematic review. Int J Equity Health 17:37, 2018 10.1186/s12939-018-0748-6 PMC5863824 29566721

[18] Ohmann C, Kuchinke W, Canham S, et al: Standard requirements for GCP: Compliant data management in multinational clinical trials. Trials 12:85, 2011 10.1186/1745-6215-12-85 PMC3074516 21426576

[19] Marchesi E, Monti M, Nanni O, et al: New requirements for phase I trials: A challenge for Italian clinical research. Tumori 104:15-21, 2018 10.5301/tj.5000668 28799641

[20] Gülmezoglu AM, Pang T, Horton R, et al: WHO facilitates international collaboration in setting standards for clinical trial registration. Lancet 365:1829-1831, 2015 10.1016/S0140-6736(05)66589-0 15924966

[21] Viergever RF Li K Trends in global clinical trial registration: An analysis of numbers of registered clinical trials in different parts of the world from 2004 to 2013 BMJ Open 5 e008932 2015 10.1136/bmjopen-2015-008932 PMC4593134 26408831

[22] Haeusler JMC: Certification in good clinical practice and clinical trial quality: A retrospective analysis of protocol adherence in four multicenter trials in the USA. Clin Res Regul Aff 26:20-23, 2009

[23] Morgan C: Clinical trials: Collaboration is the secret to successful outsourcing. www.pharmaphorum.com/r-d/views-analysis-r-d/clinical-trials-collaboration-secret-successful-clinical-outsourcing

[24] ClinicalTrials.gov: Study Map–Africa. https://clinicaltrials.gov/ct2/search/map?map=AF

[25] Association of Clinical Research Organizations. www.acrohealth.org

[26] Morgan C: CRO-sponsor partnerships: 10 tips for enabling better CRO and sponsor collaborations. https://www.contractpharma.com/issues/2016-10-01/view_features/cro-sponsor-partnerships

